# An Ecologically Valid, Longitudinal, and Unbiased Assessment of Treatment Efficacy in Alzheimer Disease (the EVALUATE-AD Trial): Proof-of-Concept Study

**DOI:** 10.2196/17603

**Published:** 2020-05-27

**Authors:** Neil William Douglas Thomas, Zachary Beattie, Jennifer Marcoe, Kirsten Wright, Nicole Sharma, Nora Mattek, Hiroko Dodge, Katherine Wild, Jeffrey Kaye

**Affiliations:** 1 Bruyère Research Institute Ottawa, ON Canada; 2 Department of Medicine University of Ottawa Ottawa, ON Canada; 3 Department of Neurology Oregon Health and Science University Portland, OR United States; 4 Department of Neurology Department of Veterans Affairs VA Medical Center Portland, OR United States; 5 Department of Neurology University of Michigan Ann Arbor, MI United States

**Keywords:** mild cognitive impairment, Alzheimer disease, mobile health, clinical trial, health information technology

## Abstract

**Background:**

The current clinical trial assessment methodology relies on a combination of self-report measures, cognitive and physical function tests, and biomarkers. This methodology is limited by recall bias and recency effects in self-reporting and by assessments that are brief, episodic, and clinic based. Continuous monitoring of ecologically valid measures of cognition and daily functioning in the community may provide a more sensitive method to detect subtle, progressive changes in patients with cognitive impairment and dementia.

**Objective:**

This study aimed to present an alternative trial approach using a home-based sensing and computing system to detect changes related to common treatments employed in Alzheimer disease (AD). This paper introduces an ongoing study that aims to determine the feasibility of capturing sensor-based data at home and to compare the sensor-based outcomes with conventional outcomes. We describe the methodology used in the assessment protocol and present preliminary results of feasibility measures and examples of data related to medication-taking behavior, activity levels, and sleep.

**Methods:**

The EVALUATE-AD (Ecologically Valid, Ambient, Longitudinal and Unbiased Assessment of Treatment Efficacy in Alzheimer’s Disease) trial is a longitudinal naturalistic observational cohort study recruiting 30 patients and 30 spouse coresident care partners. Participants are monitored continuously using a home-based sensing and computing system for up to 24 months. Outcome measures of the automated system are compared with conventional clinical outcome measures in AD. Acceptance of the home system and protocol are assessed by rates of dropout and protocol adherence. After completion of the study monitoring period, a composite model using multiple functional outcome measures will be created that represents a behavioral-activity signature of initiating or discontinuing AD-related medications, such as cholinesterase inhibitors, memantine, or antidepressants.

**Results:**

The home-based sensing and computing system has been well accepted by individuals with cognitive impairment and their care partners. Participants showed good adherence to the completion of a weekly web-based health survey. Daily activity, medication adherence, and total time in bed could be derived from algorithms using data from the sensing and computing system. The mean monitoring time for current participants was 14.6 months. Medication adherence, as measured with an electronic pillbox, was 77% for participants taking AD-related medications.

**Conclusions:**

Continuous, home-based assessment provides a novel approach to test the impact of new or existing dementia treatments generating objective, clinically meaningful measures related to cognition and everyday functioning. Combining this approach with the current clinical trial methodology may ultimately reduce trial durations, sample size needs, and reliance on a clinic-based assessment.

**International Registered Report Identifier (IRRID):**

DERR1-10.2196/17603

## Introduction

### Background

The current clinical trial methodology for testing dementia treatments relies on the time-honored approach of assessing enrolled individuals with a combination of self-report measures (eg, function, mood, adverse events), cognitive and physical function tests (eg, psychometric batteries, timed walks), and biomarkers (eg, neuroimaging-, cerebrospinal fluid-, plasma-based). These measures are typically collected at a baseline visit, followed by randomization of patients to a placebo or treatment arm. Patients are sent home until their next appointment, which may occur at varying time intervals depending on the phase and design of the study. In cases where follow-up is frequent (eg, every 2 weeks), the protocol needs to be modified to cover information carry-over, including practice effects, especially with regard to cognitive tests. Recency effects are also a particular concern, considering that people tend to report what they most recently experienced in the last few days as opposed to the overall quality of change for the entire period or may forget events which occurred during the period closest to the last visit. Across a wide range of behaviors and activities, self-report assessments have been shown to have weak correlations with objective measures [1-4]. In general, the amount of information that can be obtained is restricted by limits on how much testing a patient may be reasonably asked to complete at a single appointment, and by the frequency of appointments as the accuracy of information gained decreases as the testing intervals become more widely dispersed. In all cases, key data related to cognition and functions are rarely ecologically valid. Patients are asked to perform tasks that they typically never do in real life (eg, memorize a list of words, copy figures) or to describe how well they perform a task at home, although it may vary from the reported actual daily performance on those tasks.

The limitations of such an assessment paradigm result in data that is inherently variable, episodic, and proxy based. The cardinal features of change in patients with mild cognitive impairment (MCI) and early Alzheimer disease (AD) are a slow decline in cognition and function punctuated with acute, unpredictable events. This trajectory is challenging to assess with conventional tools and methods that lack sensitivity to subtle changes. Thus, for definitive efficacy trials, large samples followed for long periods of time are needed to determine if there is a meaningful change in cognition or function. In earlier phase trials, it is generally not possible to detect a clinical signal of change in these patients unless the treatment has a substantial effect size.

This state of affairs may be transformed by fundamentally changing the assessment paradigm [5-9]. If data can be collected continuously as opposed to episodically and infrequently, then the data lends itself to improving the precision of the estimate of the trajectory of change (ie, the slope of a line composed of only a few points is less certain than a line composed of hundreds or thousands of points) as well as intraindividual estimates of change (as opposed to the conventional group change dichotomy) [10]. High-dimensional, high-frequency data capture can be achieved by taking advantage of advances in in-home remote sensing, pervasive computing, and high dimensional data analytics. The objective sensed data also provides outcomes that are ecologically valid with immediate tangible clinical meaning. These outcome metrics collectively referred to as *digital biomarkers* include precise, time-stamped measures of physical activity, medication-taking behavior, sleep, socialization, and everyday cognitive function (eg, using a computer, driving). In addition, the approach employs relatively frequent (weekly) direct queries via email regarding internal states that inherently require direct reporting (eg, pain, mood states) as well as the opportunity to capture adverse events and health economic data (eg, falls, emergency department visits, clinic appointments).

### Objectives

Over the past decade, these digital biomarkers have been studied in relevant populations (healthy elderly and those with early MCI), demonstrating that they are sensitive to change and that the technology to capture these changes is feasible to deploy in older adults’ homes [7,8]. However, the specific use of this multisensor methodology in dementia-specific clinical trials is yet to be evaluated. To begin to understand how these technologies and digital biomarkers may be best employed in dementia clinical trials, we established a longitudinal research study to examine the relative feasibility and sensitivity of this approach in patients taking typical symptomatic treatments for AD (eg, cholinesterase inhibitors and other central nervous system active medications). This study, EVALUATE-AD (Ecologically Valid, Ambient, Longitudinal and Unbiased Assessment of Treatment Efficacy in Alzheimer’s Disease), is currently underway to determine the feasibility of capturing these more continuous and objective everyday measures at home, to assess the comparability of these novel measures to conventional outcome metrics, and to develop a composite model from these functional measures that can detect changes related to initiating and discontinuing common treatments employed in AD-related care. This paper describes the methodology behind the assessment protocol, presents preliminary results of feasibility measures, and provides examples of preliminary data from home-based system sensors.

## Methods

### Study Design

EVALUATE-AD is a longitudinal, naturalistic observational cohort study. Thirty patients and 30 spouse coresident care partners (a total of 60 participants in 30 households) will be enrolled and monitored continuously for up to 24 months with the home-based computing and sensor system. The participants are recruited from an existing cohort of patients followed at the National Institute on Aging (NIA)−Layton Oregon Aging and Alzheimer’s Disease Center (OADC). Additionally, new patients seen at the Aging and Alzheimer’s clinic and participants referred from community physicians are enrolled if they meet the inclusion criteria. All participants sign informed consent forms (Oregon Health and Science University, OHSU Institutional Review Board number 16515).

Participants with MCI or AD living in the Portland metropolitan and surrounding areas, together with a coresident considered as a care partner are invited to participate in the study. The inclusion criteria for the participants with cognitive impairment and their coresidents include the following: NIA and the Alzheimer’s Association clinical criteria for MCI [11] or probable AD [12] and have a Mini-Mental State Examination (MMSE) [13] score of 15 to 30, inclusive; the coresident care partner is functionally independent and has an MMSE of 24 to 30, inclusive; any gender; aged 50 to 90 years; consents to enrollment in the protocol; The coresident care partner is computer literate, defined as being able to send and receive an email; the household owns and uses a desktop or laptop computer; households have a reliable, broadband internet connection; and live in a larger than 1-room apartment.

The exclusion criteria are as follows: Significant neurologic diseases other than MCI or early AD, such as multi-infarct dementia or vascular cognitive impairment, Parkinson’s disease, normal pressure hydrocephalus, brain tumor, or a history of significant head trauma with subsequent persistent neurologic deficits; major psychiatric disorders such as major depression, bipolar disorder (Diagnostic and Statistical Manual of Mental Disorders, 4th Edition; DSM-IV criteria) within the past year, or history of schizophrenia (DSM-IV); psychotic features, agitation, or behavioral problems within the last 3 months, which could lead to difficulty complying with the protocol; history of alcohol or substance abuse or dependence within the past 2 years (DSM-IV criteria); any uncontrolled medical condition that is expected to preclude completion of the study, such as late-stage cancers; and more than 2 people live in the participant’s residence (overnight visitors are acceptable).

Participants have dementia screening laboratory studies (complete blood count, chemistry panel, thyroid function, vitamin B-12), and brain imaging (magnetic resonance imaging or computed tomography) as part of their initial diagnostic work-up. An in-home screening visit is conducted by a research coordinator where consent is obtained, self-report questionnaires are completed, and neurocognitive tests are administered. A baseline assessment is then performed by a clinician at the participants’ residence with a physical and neurological exam and neurocognitive tests. At 12 months and at the end of the study, the self-report questionnaires, physical and neurological exam, and neurocognitive tests are repeated during separate home visits by the research coordinator and clinician. The full assessment protocol, including baseline and follow-up assessments are shown in Table 1.

**Table 1:** Study schedule of assessments.

Assessment type	Week 0 (screening visit)	Week 0 (baseline assessments)	Week 1 (technology installation visit)	Week 52 (12-month assessments)	Week 104 (24-month assessments)
Consent	X^a^	—^b^	—	—	—
Personal and Family History Questionnaire	X	—	—	X	X
Subject Memory and Health Rating	X	—	—	X	X
MMSE^c^ [13]	X	—	—	X	X
ADAS-Cog^d^ [14]	X	—	—	X	X
Geriatric Depression Scale [15]	X	—	—	X	X
ISAAC^e^ Technology Use Survey	X	—	—	X	X
Handedness Inventory	X	—	—	—	—
Technology and Computer Experience and Proficiency Questionnaires	X	—	—	X	X
Functional Assessment Questionnaire [16]	X	—	—	X	X
Neuropsychiatric Inventory Questionnaire [17]	X	—	—	X	X
Zarit Burden Interview–Short [18]	X	—	—	X	X
Pittsburgh Sleep Quality Index [19]	X	—	—	X	X
WRAT^f^ reading level	X	—	—	—	—
Neurobehavioral Cognitive Status Examination [20]	—	X	—	X	X
Clinical Dementia Rating [21]	—	X	—	X	X
Neurological examination	—	X	—	X	X
Modified Unified Parkinson Disease Rating Scale [22]	—	X	—	X	X
Medical history and comorbid conditions	—	X	—	X	X
Tinetti gait	—	X	—	X	X
Tinetti balance	—	X	—	X	X
Sensor system installation	—	—	X	—	—
ORCATECH^g^ Health and Life Activity Form	—	—	Assessed weekly	Assessed weekly	Assessed weekly
Total activity: mobility, steps, gait speed, and time in locations	—	—	Assessed continuously	Assessed continuously	Assessed continuously
Socialization and caregiving: time out, time alone or with partner, and time on internet	—	—	Assessed continuously	Assessed continuously	Assessed continuously
Medication taking: adherence (also weekly)	—	—	Assessed continuously	Assessed continuously	Assessed continuously
Cognition: computer activity, time on; session times, and complete forms	—	—	Assessed continuously	Assessed continuously	Assessed continuously
Sleep: time up, time in bed, times up at night, restlessness, and sleep latency	—	—	Assessed daily	Assessed daily	Assessed daily
Physiology: BMI and pulse	—	—	Assessed daily	Assessed daily	Assessed daily

^a^X: Assessment performed at this visit.

^b^Assessment not performed at this visit.

^c^MMSE: Mini-Mental State Examination.

^d^ADAS-Cog: Alzheimer Disease Assessment Scale–Cognitive Subscale.

^e^ISSAC: Intelligent Systems for Detection of Aging Changes.

^f^WRAT: Wide Range Achievement Test.

^g^ORCATECH: Oregon Center for Aging and Technology.

### Components of the Assessment System

**Figure 1:**
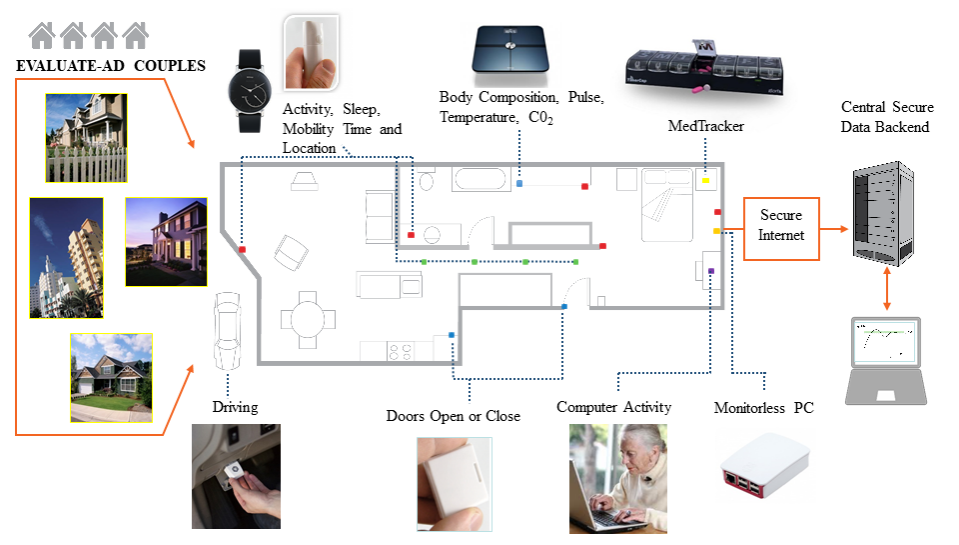
Schematic of the home-based sensor system. EVALUATE-AD: Ecologically Valid, Ambient, Longitudinal and Unbiased Assessment of Treatment Efficacy in Alzheimer’s Disease.

After the screening and baseline clinician visits are complete, the sensor system is deployed at the participants’ residence by a technology deployment field team according to the established Oregon Center for Aging and Technology (ORCATECH) Life Laboratory protocols [7-9] and the Collaborative Aging Research using Technology (CART) initiative [23]. Initial data are recorded with regard to the layout of the home to label the use of various spaces (eg, kitchen, bathroom, bedroom, etc). To facilitate deployment of the system in the community, where each home typically has a unique layout, a tablet-based graphing tool is used to automatically record where various sensors are located and their physical adjacencies to other sensors. A schematic of the overall home-based setup is shown in Figure 1; specific details of each component are described in Multimedia Appendix 1 [24-34] and are available on the CART initiative website [35].

The components are described briefly as follows:

1. Hub computer:
  A monitorless computer (Raspberry Pi) functions as a data hub for all the sensors. Data are collected via standard wireless communications protocol (eg, Bluetooth, Zigbee, Wi-Fi) and transferred securely to servers at OHSU.
2. Activity sensing:
  Passive infrared (PIR) motion sensors using the Zigbee wireless communication protocol (NYCE Control) are placed in each room in the home and sense participants’ motion at home and transitions between rooms. A line of four PIR sensors with more restricted fields of view are placed on the ceiling in an area where the participant walks regularly to detect walking speed. Each participant will also wear an activity-monitoring wristwatch (Withings Steel) to measure individual mobility and sleep measures.
3. Medication-taking behavior:
  An electronic pillbox (TimerCap iSort) records the times when specific lids (marked by the days of the week) are opened and closed. The electronic pillbox is provided to the participants with cognitive impairment to track their medication usage. Care partners do not use the pillbox. However, care partners can assist or remind the patient to take medications if this is part of their normal routine.
4. Physiological monitoring:
  Participants are asked to weigh themselves daily using a digital bioimpedance scale (Withings Body Cardio).
5. Driving assessment:
  An on-board telematic device (Automatic Pro) records data on multiple aspects of driving behavior and connects to the on-board diagnostic (OBD-II) port in each participant’s vehicle.
6. Computer-based monitoring:
  WorkTime software (Nestersoft) is installed on the computers of each participant, which records data on computer use (eg, time spent using the computer, number of sessions on the computer per day).

### Medication Changes

To provide a conventional measure of changes in cognition that occur when patients transition on or off AD-related medications (cholinesterase inhibitors, memantine, antidepressants, hypnotics), the Telephone Interview for Cognitive Status (TICS) [36] is administered to participants within 1 week of a change in these medications and then subsequently at 6 and 12 weeks. Scores from the TICS are highly correlated with the MMSE [36]. Prior studies of cholinesterase inhibitors in individuals with AD administered the MMSE at baseline, 6, and 12 weeks and found a significant difference in MMSE scores at 12 weeks [37,38]. Changes in medication are identified using the weekly self-report survey, and an alert is sent from the ORCATECH home-participant management system to a research coordinator when participants indicate a medication change.

### Analytic Considerations

This study is a proof of concept designed to construct a composite model of sensor-derived outcome measures that correlate with changes in conventional cognitive test scores seen when individuals start or stop cholinesterase inhibitors, memantine, or other medications, such as antidepressants, that are commonly used for managing AD. As this is an observational study, participants with MCI and AD are followed longitudinally, but medication changes are not dictated or restricted by the study; the participants’ primary clinician prescribes these medications according to their practice. Therefore, participants may start, increase the dose, discontinue, or never be on AD-related medications. The Alzheimer Disease Assessment Scale–Cognitive Subscale (ADAS-Cog 11) was chosen for comparison to previous trials that found significant improvements in cognitive function with cholinesterase inhibitors [37-40] and memantine [41,42] relative to placebo. The ADAS-Cog is performed at baseline, 1 year, and 24 months (study end). The continuous sensor-based measures will be compared with the ADAS-Cog test scores. The effect of changes in dementia-related medications will be analyzed in a subset of participants where those changes occur. Our hypothesis is that changes in medications can be detected by high frequency, in-home monitored data with higher sensitivity (ie, high signal-to-noise ratio) than cognitive test scores, based on a previous study where we could reduce intraindividual variability and thereby reduce the required sample size [10].

### Analysis

#### Feasibility Measures (Adherence and Dropout)

The first objective of this study is to assess the feasibility of using home-based pervasive computing systems to identify changes in meaningful outcomes in patients across the spectrum of MCI through early AD. Accordingly, the focus of analysis is on measures of adherence, retention, and report of experience with the technologies and protocol. Primary measures are the percentages of completed weekly web-based health and activity forms and dropout at 24 weeks and at the end of the study. Criterion measures are >80% adherence to completion of the weekly web-based survey and 0 dropout (for nonmedical reasons). In addition, information on each participant’s experience with respect to the home sensor will be collected using a modified home monitoring technology attitudes and beliefs survey administered at the study end or early discontinuation.

#### Description of Sensor-Based Measures

The measures from nine individual functional/health domains evaluated are summarized in Table 2. The sensors collect data on a daily or continuous basis that provides information on the core functions and measures. Sensor-derived outcome measures from each domain will be compared with the corresponding conventional assessment measures in subsequent analyses at the completion of study data collection.

**Table 2:** Core functions and measures collected and types of sensors used to collect data. Metrics may be event driven (eg, medication taking) or unscheduled (eg, minutes to days of total activity).

Core functions and measures (continuous, daily, or weekly)	Sensors or devices used	Conventional assessment measures (at baseline, 12- and 24-months follow-up)
Physical capacity and personal mobility: Total daily activity, number of room transitions, median weekly walking speed from multiple daily walks, daily steps, and time out of home	PIR^a^ motion sensors and door contact sensors; wearable activity tracking wristwatch	Walking speed (with a stopwatch). Self-report of activity from the OADC^b^ Personal and Family History Questionnaire (Paffenbarger scale [43], for example, *estimate how many hours per day you spend in moderate activity*)
Sleep and nighttime behavior: Time of awakening in the morning, time spent in bed at night, wake after sleep onset, times up at night, and sleep latency	PIR motion sensors; wearable activity tracking wristwatch	Pittsburgh Sleep Quality Index and Sleep Disturbance Symptom Questionnaire [19] (part of the OADC Personal and Family History Questionnaire)
Physiologic health: daily BMI, pulse	Biofunction scale (AM pulse)	Vital signs (height, weight, pulse)
Medication adherence: Percentage of doses missed in a 7-day period, relative to the prescribed schedule.	Electronic pillbox	Self-report of adherence to medication-taking regimen (visual analog scale: ranging from 0% to 100%)
Socialization and engagement: Time out of home, time alone or with spouse, and computer activity	PIR motion sensors, contact sensors; wearable activity tracking wristwatch; personal computer	Self-report of eight social activities from the OADC Personal and Family History Questionnaire (eg, how often do you have visitors: rarely/never, daily, weekly, monthly, yearly)
Cognitive function: Time to complete online tasks (eg, weekly web-based online health forms), mouse movements, prospective memory for medication, and AM weighing protocol.	Personal computer or tablet; electronic pillbox; biofunction scale.	ADAS-Cog^c^ 11 score ^[14]^, MMSE^d^ score [13], NCSE^e^ scores [20], TICS^f^ [36] (completed if participant has an AD^g^-related medication change)
Community mobility: Driving time and distance driving, hard braking, hard accelerations, and most frequent locations out of home	Home sensors (exit door contact sensors); automobile data port telematic sensor	FAQ^h^ [16] rating of ability: traveling out of neighborhood, driving, arranging to take buses
Health and life events: online self-report (ie, ER^i^, doctor, or hospital visits, home visitors, mood, pain, loneliness, falls, injuries, change in home space, home assistance received, change in medications)	Personal computer or tablet (online reporting)	Mood: Geriatric Depression Scale (15-item) [15] and Neuropsychiatric Inventory [17]; self-report of health events from the OADC Personal and Family History Questionnaire
Care partner engagement: Time alone or time with cognitively impaired partner, time in bathroom together	PIR motion sensors; door contact sensors; wearable activity tracking wristwatch	Zarit Caregiver Burden Scale [18]

^a^PIR: passive infrared.

^b^OADC: Oregon Aging and Alzheimer’s Disease Center.

^c^ADAS-Cog: Alzheimer Disease Assessment Scale–Cognitive Subscale.

^d^MMSE: Mini-Mental State Examination.

^e^NCSE: Neurobehavioral Cognitive Status Examination.

^f^TICS: Telephone Interview for Cognitive Status.

^g^AD: Alzheimer disease.

^h^FAQ: Functional Assessment Questionnaire.

^i^ER: emergency room.

## Results

### Participant Characteristics

Thirty homes have been enrolled and had the home assessment system installed (Figure 1), as of February 2020. Here, we present the preliminary data from the first 10 dyads with over first 6 months of monitoring after enrollment, composed of 5 participants with AD and 5 participants with MCI and their respective care partners (20 participants total). Participants with cognitive impairment were, on average, 74.7 years old with 17.7 years of education (Table 3). Mean scores on the MMSE were 24.9 and 13.7 on the ADAS-Cog. Care partners were, on average, 71.1 years old with a mean MMSE score of 29.7. The mean total duration of monitoring for the first 10 homes was 14 months.

**Table 3:** Demographics for participants from 10 homes (N=20).

Baseline variable	Patient (n=10)	Care partner (n=10)
Age (years), mean (SD)	74.7 (7.5)	71.1 (8.5)
Female, n (%)	2 (20)	8 (80)
Education (years), mean (SD)	17.7 (3.0)	16.3 (2.4)
MMSE^a^, mean (SD)	24.9 (5.0)	29.7 (0.7)
ADAS-Cog^b^ (n=9), mean (SD)	13.7 (10.4)	N/A^c^
CDR^d^, mean (SD)	0.7 (0.2)	N/A
GDS^e^, mean (SD)	2.2 (2.3)	1.5 (1.6)
ZBI-12^f^, mean (SD)	N/A	10.0 (7.5)
NPI-Q^g^, mean (SD)	3.3 (3.2)	N/A
FAQ^h^, mean (SD)	8.4 (9.5)	N/A
Dementia-related medications, n (%)	6 (60)	N/A

^a^MMSE: Mini-Mental State Examination.

^b^ADAS-Cog: Alzheimer Disease Assessment Scale–Cognitive Subscale.

^c^N/A: not applicable.

^d^CDR: Clinical Dementia Rating.

^e^GDS: Geriatric Depression Scale.

^f^ZBI-12: Zarit Burden Interview–Short.

^g^NPI-Q: Neuropsychiatric Inventory Questionnaire.

^h^FAQ: Functional Assessment Questionnaire.

### Recruitment

**Figure 2:**
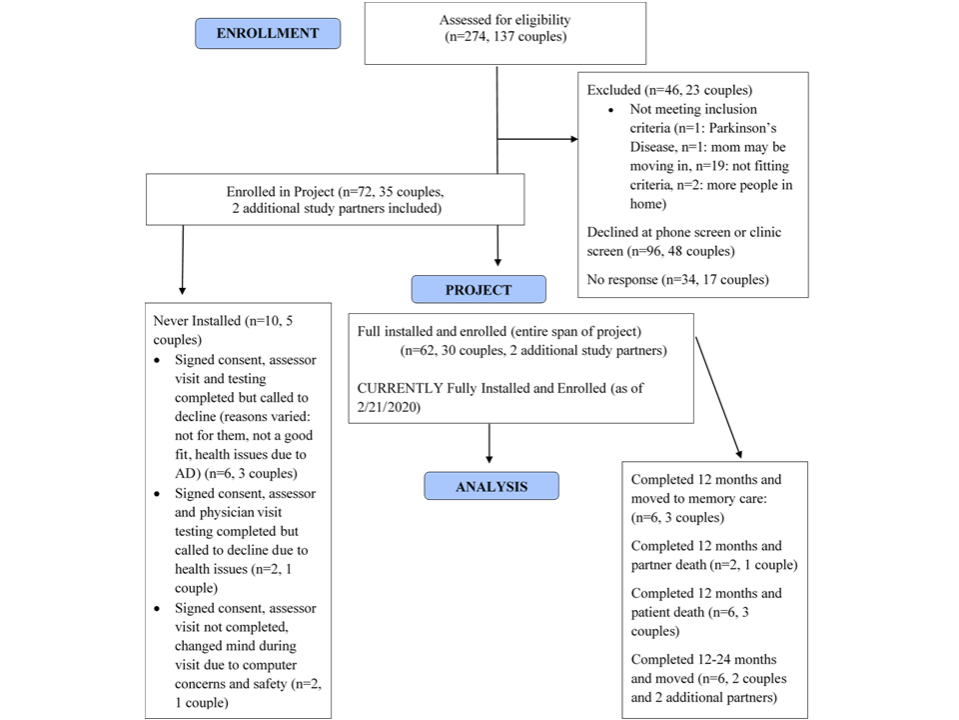
Participant enrollment and follow-up summary. Two homes were enrolled with a third additional study partner in the home, who also wore an activity monitoring wristwatch. AD: Alzheimer disease.

The screen failure rate was approximately 68.04% (132/194 individuals) for eligible participants (Figure 2). A total of 274 participants were assessed for eligibility, with 46 not meeting criteria and 34 not responding to messages left about participation in the trial. Other individuals who were contacted declined participation for a variety of reasons. The majority of individuals that declined indicated they were not interested in participating in a clinical trial at the time of contact. Some individuals were more interested in participation in an interventional trial, and others declined because their study partner did not agree to be involved in the trial. The installation of a home assessment system or having to wear an activity monitoring wristwatch was offered as another reason for declining participation in the study.

### Feasibility Measures

#### Acceptance of the Home Assessment System

The home-based pervasive computing system is well tolerated by participants. There have been no withdrawals from the study after the system has been deployed in the home. Exit survey responses were available from the care partners of the two homes that completed the study due to the individual with cognitive impairment transitioning to long-term care. The exit surveys are shown in Multimedia Appendices 2 and 3. Both care partners strongly agreed with the statements *I do not mind being monitored unobtrusively in my home*, and *I did not find the sensor system was an extra source of stress.*

#### Adherence

Adherence to completion of the weekly web-based health survey was 75% for participants with cognitive impairment (n=6, independently completing on the web) and 84% for care partners (n=10; Table 4), with the longest enrollment in the study being 396 days. The completion rate was good for the care partners; however, the completion rate for participants with cognitive impairment was slightly lower than the criterion rate. A total of 4 of the 5 participants with AD required assistance with the completion of the survey each week from their care partner.

**Table 4:** Summary of sensor-based measures in patient participants and care partners.

Sensor system outcome measure	Patient (n=10)	Care partner (n=10)
Follow-up time (months), mean (SD)	14.6 (3.0)	13.9 (4.1)
Mean daily total steps, mean (SD)	3709 (3245)	4089 (2230)
Daily watch compliance (%), mean (SD)	75 (15)	72 (12)
Mean nightly sleep time (hours), mean (SD)	7.2 (0.8)	7.8 (0.6)
Nightly watch compliance (%), mean (SD)	60 (23)	65 (14)
Electronic pillbox compliance (%; n=6), mean (SD)	77 (26)	N/A^a^
Independently completing online weekly health form, n	6	10
Weekly health form compliance (%), mean (SD)	75 (27)	84 (16)

^a^N/A: not applicable.

#### Instances of Missing Data

A few technical issues were encountered during the enrollment and data collection of the first few participants. This was mainly due to a major upgrade in the home monitoring system that included, in part, the addition of new devices (eg, activity monitoring wristwatch and new electronic pillbox). These issues were quickly identified and resolved using a series of software and firmware updates.

### Sensor-Based Outcome Measures

Table 4 shows a summary of a sample of sensor-based outcome measures comparing care partners with participants with cognitive impairment.

#### Medication-Taking Behavior

**Figure 3:**
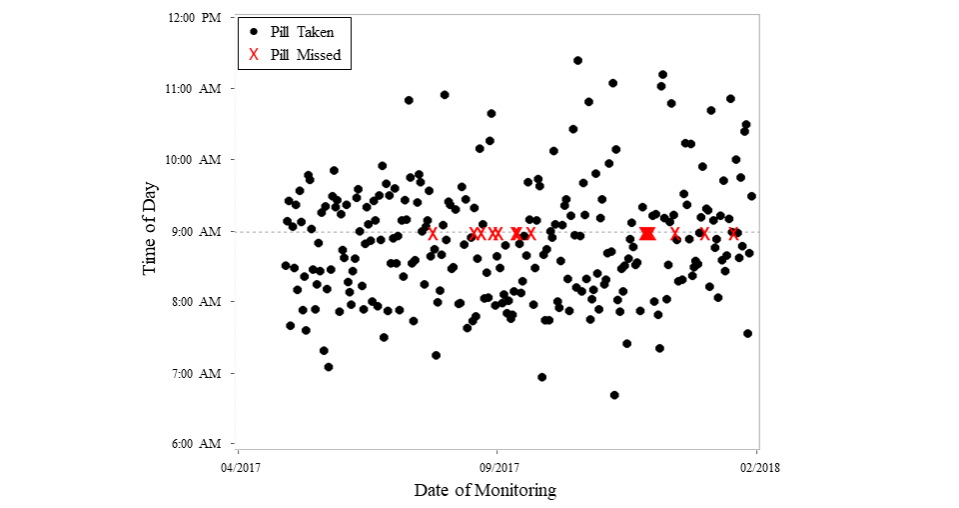
Time of day that medication was taken for each day over 7 months of monitoring by a participant with mild Alzheimer disease. The dots indicate the times at which the pill was taken, and an X indicates when a pill was missed. Overall, participant adherence was 94% over 9 months.

Of the 10 participants with cognitive impairment, 6 (5 with AD, 1 with MCI) were taking AD-related medications (cholinesterase inhibitors, memantine, antidepressants, or sleep aids) and using the electronic pillbox. Overall compliance for the group was 77% (Table 4). Figure 3 shows adherence for a single participant over 7 months for a once-daily medication (venlafaxine).

#### Activity Sensing and Sleep Behavior

Preliminary data collected from the activity monitoring are presented from a mean of 14.6 months of monitoring in participants with cognitive impairment. In this sample, participants with cognitive impairment (n=10) had a mean step count of 3709 and a mean total sleep time of 7.2 hours per night. Care partners (n=10) had a mean step count of 4089 and a mean total sleep time of 7.8 hours per night. Compliance to wearing the watch ([number of days with watch data]/[total number of days]×100) for both groups is shown in Table 4.

#### Changes in Medications

**Figure 4:**
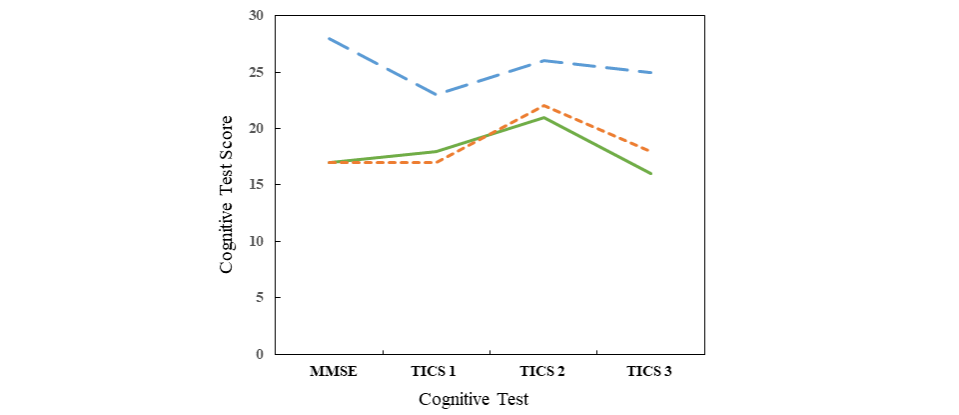
Cognitive test scores in the 3 participants with medication changes. The MMSE was completed at the baseline study visit. The Telephone Interview for Cognitive Status were completed over the phone after a change in medication and subsequently at 6 and 12 weeks. MMSE: Mini-Mental State Examination; TICS: Telephone Interview for Cognitive Status.

Changes in AD-related medications occurred in 3 participants. The changes were all related to antidepressant medications used to treat behavioral symptoms associated with AD. Two participants had the dose of their medication increased and 1 was started on a new antidepressant medication. Figure 4 shows the results of TICS at the time of medication change (TICS 1), at 6 weeks (TICS 2), and at 12 weeks (TICS 3). In addition to the cognitive testing performed after medication changes, the weekly health report form also collects information that may be relevant to medications treating behavioral and psychiatric symptoms of dementia. Participants are asked if they have felt *blue* or *lonely* in the past week. In 1 participant, reports of feeling *blue* decreased from 33% (7/21) of weekly responses before the medication change to 10% (3/30) afterward, and reports of feeling *lonely* decreased from 24% (5/21) to 3% (1/30). In the other 2 participants, reports of feeling *blue* or *lonely* did not change significantly. In the second participant, there were no reports of feeling *blue* and only one report of feeling *lonely* after the medication change. In the third participant, there was one report of feeling *blue* before the medication change, with none afterward, and only one report of feeling *lonely* after the medication change.

## Discussion

### Initial Findings

The EVALUATE-AD trial aims to determine the feasibility of detecting changes in everyday health and functional domains that are related to cognitive impairment in individuals with MCI and AD. In order to properly utilize remote sensing approaches in clinical trials, potentially more sensitive, objective, and ecologically valid measures⸺digital biomarkers⸺need to be longitudinally acquired and analyzed in real-world environments. Although individuals with MCI have been studied with home-based sensing systems for extended periods of time [44,45], people with early AD and their care partners have not. The collection of digital biomarkers in more natural settings provides the opportunity to collect data on novel outcomes related to daily functioning that cannot be ascertained with conventional clinic-based methods. Additionally, the data collection occurs unobtrusively and with little involvement of the participants, thereby avoiding the addition of potential stress and burden to individuals with cognitive impairment and their care partners.

Preliminary results from this study demonstrate that the deployment of the home-based computing and sensing system is well received by participants. There has been no dropout after study enrollment. Adherence to completion of the weekly health survey is above the expected criterion value for care partners, but slightly below the criterion for individuals with cognitive impairment. The difference between groups may be in part related to the need for assistance in completion of the form in some individuals with AD. Outcome metrics comprising multiple functional and health-related domains are being collected and analyzed from multiperson homes. Examples from preliminary data show how medication adherence, activity levels, and sleep behavior can be collected longitudinally by the home-based system. The use of an electronic pillbox has potential limitations, as the opening and closing of a daily compartment does not guarantee that the medication was ingested. However, daily monitoring of medication-taking behavior with this sensor should provide greater accuracy than the current practice of relying on study participants to bring unused medication to study visits for tabulation. Compliance with wearing the activity-monitoring wristwatch was higher during the day than at night and was collected for 60% of the nights in participants with cognitive impairment. This demonstrates the potential shortcoming of wearable technologies in everyday long-term use. Participants may not feel comfortable wearing the watch during sleep. Additionally, if the device is removed during the day, individuals may forget to put it back on. The activity watch provides the advantage of detecting activity levels even when the participant is outside of the home, but for monitoring sleep, unobtrusive sensors (eg, PIR sensors and movement-sensitive bed mats) may provide more reliable methods for longitudinal monitoring.

Technical issues that arose initially during the study demonstrated problems that can arise as new sensors are integrated into a platform. To ensure that all sensors were functioning, modifications to the alert system in the home monitoring platform were designed. An automated program was created to summarize the data from each sensor in each home on a weekly basis. Sensors that may not have collected data on a specific day still generate a regular *check-in* signal to ensure that they are functioning properly. This system also provides frequent data reviews to identify issues that arise with data collection as early as possible. Any issues that were detected by the program were identified by the study coordinator and the technology field team for the study, and a solution to the problem was provided either remotely or with a home visit if necessary. The technical solutions to these issues can be applied as new sensors continue to be integrated into research platforms and will help improve the reliability of data collection and prevent loss of data.

### Future Analysis

The second objective of the project is to compare the outcome measures of the automated system in different functional and health domains with conventional clinical outcome measures in AD. As part of the evaluation of these novel approaches, comparison to current standards need to be conducted, and three approaches will be applied. Data from the continuous sensor-based measures will be aggregated from 2-month periods anchored on the date of conventional measure acquisition. This is done because the frame of reference of the conventional measure comparator is restricted to a single day and is a method used in previous studies [24]. For these comparisons, simple correlations will be calculated between the objective, continuous sensor-derived variables, and the conventional test domains in the total cognitively impaired sample regardless of diagnosis and then in a secondary analysis dividing the group into MCI and early AD. The second approach examines the trajectories of change in continuously collected sensor-based measures, using a previously established procedure to determine these trajectories [10]. A subject-specific distribution is calculated for each metric using the data collected during the first month, and an individual-specific threshold of low and high activity is created. The change (or shift) in individual-specific distributions over time can then be examined by tracking how often individuals move below or above their own threshold determined at baseline (ie, during the first 3 months). This approach, which utilizes individual-specific distributions instead of group means, was found to be sensitive to changes even among those with presymptomatic MCI, where detection of change is often quite difficult. Finally, using generalized mixed effects models, the likelihood of having low functional days that differs by diagnostic group (MCI or early AD) and medication status (eg, taking anticholinesterase medication vs not taking them) is determined. Before applying the above approach, we ensure that the trajectories for each metric are reasonable in terms of ranges, direction, and the amount of change using conventional approaches, such as examination of spaghetti plots, linear mixed effects models with or without nonlinear terms, and latent trajectory models (an approach successfully employed in previous work [25]).

The third goal of the project is to develop an objective behavioral-functional signature of patients on cholinesterase inhibitors and related therapies. This measure will be derived from a composite model composed of sensor-based outcome measures that are found to be significant in detecting differences in trajectories by cognitive impairment group as well as those on or off symptomatic AD treatments. The ultimate goal is to examine whether those initially without treatment or adjustment to treatment show changes (ie, improvement) in the derived digital composite score over time when they are on the medication. High-frequency, multidomain data afforded by the pervasive computing environment deployed affords the ability to identify contrasting dynamic changes in relevant functions between different pharmacologic agents. Those relevant to current, approved therapy form a baseline of activities and behaviors to contrast for future trials. This pharmacologic behavioral fingerprinting and, ultimately, the generation of more meaningful composite measures can be generalized to future randomized control trials using new agents. This objective is not the focus of this preliminary report and will be reported in a subsequent publication once data collection for the trial is complete.

Although a focus of this research is to detect treatment-specific changes, the sample size is small, and not all participants in the study will transition on or off a cholinesterase inhibitor, memantine, or a symptom-management medication. Nevertheless, we anticipate that a composite digital biomarker composed of multiple outcome measures derived from the home monitoring system will detect sensitive changes in the digital biomarker signal with increased statistical power. Unlike the presymptomatic subjects enrolled in prior studies [7], the MCI and AD patients recruited in this study are anticipated to experience greater cognitive decline (ie, MMSE declines by 0.02 points per year among presymptomatic subjects or over 5 years of change≈1 MMSE point), with MMSE declines of 2 or 3 points per year observed for AD patients (ie, a >10-fold faster decline) [46]. Given that we would see an approximately 8-fold steeper decline in outcomes than previously shown, using this intraindividual approach, we would achieve 80% power to detect a 30% treatment effect size with 30 subjects (20 subjects with medication and 10 subjects without) over 2 years (alpha=.05, 2-tailed). The automated sensor-based measures collected in EVALUATE-AD for up to 24 months will provide important measures of variance and trajectory of change data needed for future power estimates.

### Conclusion

The use of high-frequency, longitudinal data acquisition appears more sensitive to change than conventional, episodic in-clinic testing. The measures lend themselves to more direct translation to meaningful outcomes for patients and care partners (eg, improved mobility, computer use, better sleep, better medication adherence). These digital biomarkers can be used in combination with conventional clinical assessment methods. A behavioral-pharmacologic signature composed of multiple digital biomarkers could be used to detect changes in cognition and functional status in individuals with cognitive impairment initiating or discontinuing symptomatic treatments. This methodology has the potential to reduce the size and/or length of clinical trials by more precisely estimating the true trajectory of change in participants with high-frequency in-home data and individual-specific distributions. The ultimate goal will be to use these longitudinal and person-specific measures to more effectively test new therapeutics and guide individual responses to therapies in patients.

## Appendix

Multimedia Appendix 1 Components of the assessment system.

Multimedia Appendix 2 Participant exit survey examining attitudes and perceptions towards the home-based sensor system.

Multimedia Appendix 3 Care partner exit survey examining attitudes and perceptions towards the home-based sensor system.

## Abbreviations

AD: Alzheimer disease
ADAS-Cog: Alzheimer Disease Assessment Scale–Cognitive Subscale
CART: Collaborative Aging Research using Technology
DSM-IV: Diagnostic and Statistical Manual of Mental Disorders, 4th Edition
EVALUATE-AD: Ecologically Valid, Ambient, Longitudinal and Unbiased Assessment of Treatment Efficacy in Alzheimer’s Disease
MCI: mild cognitive impairment
MMSE: Mini-Mental State Examination
NIA: National Institute on Aging
NIH: National Institutes of Health
OADC: Oregon Aging and Alzheimer’s Disease Center
OHSU: Oregon Health and Science University
ORCATECH: Oregon Center for Aging and Technology
PIR: passive infrared
TICS: Telephone Interview for Cognitive Status
