# Supplementary material for: An Ecologically Valid, Longitudinal, and Unbiased Assessment of Treatment Efficacy in Alzheimer Disease (the EVALUATE-AD Trial): Proof-of-Concept Study
Source: JMIR Res Protoc. 2020 May 27;9(5):e17603. doi: 10.2196/17603 (PMC7287724; doi:10.2196/17603)
Supplement: Multimedia Appendix 1 [file resprot_v9i5e17603_app1.docx]

**Appendix**

**Hub Computer.** A monitorless computer (Raspberry Pi Foundation) with an Ethernet connection is used for data collection from the system devices or sensors and data transmission back to secure servers at OHSU without participant involvement. This computer functions as the data hub for all sensed data from the home. The hub computer is placed in an out-of-the-way location and configured to the specific participant and home during system installation using a laptop, tablet or smartphone (the field team brings this laptop, tablet or smartphone to the home for installation purposes only). As the data hub for the home, installed sensors and devices (described below) are configured to communicate with the hub computer. The hub computer allows for communication with the ORCATECH centralized digital home-participant management system.

**Activity Sensing.** Passive infra-red (PIR) motion sensors using the Zigbee wireless communication protocol (NYCE Control, Vancouver, BC) are strategically placed in the home. Sensors are digitally assigned to a given home during system installation, communicating with the hub computer via small transceivers (Telegesis Zigbee dongles, Silicon Labs, Austin, TX). Depending on the size of the home, one to six of these dongles are placed around the home to provide reliable sensor communication. One PIR sensor is placed per room at head height (affixed to the wall with two-sided 3M Velcro tape) to sense motion within the room and participant transitions from room to room. An in-series “Sensor Line” array of four PIR sensors, which have a more restricted fields of view and are spaced at 0.6 meter intervals is placed on the ceiling of a hallway or another area where the participant will regularly walk or pass through at a consistent pace [24]. This sensor line enables unobtrusive gathering of walking speed and variability many times per day and has been shown to detect walking speeds that differentiate MCI from normal volunteers [25]. Other metrics can be derived from these motion sensors such as presence in a room, number of room transitions, or time out of the home [26,27]. These measures (coupled with information from an activity monitoring wristwatch – see below) may be particularly relevant for caregiver interaction (e.g., calculating the time a dyad may spend in rooms together or alone). Contact sensors (NYCE Control) are placed around the home on all external doors to detect (coupled with in-room sensors) participants coming and going from the home (facilitating the determination of time out of home measurements). To capture overall individual mobility and activity, participants wear a water resistant (5ATM) Withings Steel activity monitoring wrist watch (Withings Inc.). These data are used to generate activity measures in conjunction with the passive motion PIR sensors location data noted above. Metrics include total activity, step count, sleep measures (total sleep time) and time either alone or together with co-resident by home location (in bathroom, bedroom, kitchen, living room, out of home). To enhance sustained compliance the participants wear the activity watch on the wrist that they typically wear their wrist watch (on their non-dominant wrist) [28]. Also, an automated program summarizes the data from the watch on a weekly basis. The study coordinator and technology field team for the study monitor these reports and create alerts if no activity data is being received from the watch. The watch has a battery life of up to 8 months. Batteries will be replaced prior to their proposed life expectancy by the field team or if there is indication from the participant or the data collection system that the watch is no longer functioning.

**Medication Taking Behavior – TimerCap iSort.** This is a seven-day electronic pill box that records whether or not the designated day’s compartment was opened and closed and the time(s) that it was opened each day. The iSort transmits the information wirelessly to the hub computer via BLE (Bluetooth Low Energy). The iSort provides valuable information about study medication adherence, as well as a potential indication of cognitive decline (impairment of prospective memory function) if consistency of medication-taking declines [29,30]. Participants with cognitive impairment will be asked to use the iSort for symptomatic AD therapies (cholinesterase inhibitors, memantine) and medications related to behavioral and psychiatric symptoms of dementia (anti-depressants, anxiolytics, sleep related medications).

**Physiological Monitoring.** A Wi-Fi and Bluetooth enabled digital bioimpedance scale (Withings Inc.) capable of measuring weight, body mass index (with body fat and water percentage, muscle and bone mass), and pulse (while standing on the scale) is placed on a secure surface in the bathroom. The scale transmits data via Wi-Fi to the Withings website using the hub computer as a secure access point. The cognitively impaired participant is asked to stand on the scale each morning without socks or shoes, with instructions to pause long enough to obtain the pulse measurement (typically < 20 sec following the weight measurement). In addition to the physiologic measures, the regularity of remembering to perform this prospective memory task is also measured.

**Driving Assessment.** Automatic Pro (Automatic Labs, Inc.) on-board telematic devices that connect unobtrusively to a vehicle’s On-Board Diagnostic (OBD-II) port are used to monitor driving behavior. This device is compatible with vehicles sold in the U.S. beginning with the 1996 model year. For subjects with compatible vehicles, onboard devices will be installed in participants’ vehicles. Raw trip data will be obtained electronically from each device, and transmitted via cellular link (3G wireless) to the Automatic commercial servers. Driving data is then uploaded to the ORCATECH central research database utilizing Automatic’s API. Data available for each trip includes, but is not limited to, start location, start time, end location, end time, trip distance, number of hard accelerations, number of hard brakes, and number of seconds over 70mph during the trip [31].

**Computer-Based Monitoring and Self-Report System.** Each participant uses his or her own computer. Couples may share a home computer, in which case a user login is used to identify each user. Participants are instructed to use their computers as they normally do, without specified schedules or activities. Software (WorkTime; NesterSoft Inc.) is installed on the participant’s computer allowing for the capture of multiple event types (general applications used, time on computer, number of computer sessions) [32]. The participants are asked to use individual login profiles to help collect individual computer use data. Specific content of personal data, communications such as email, or specific websites visited are not collected. In addition to this passive computer-based activity data, each week participants are sent an online weekly self-report survey via email that can be completed on any device with an internet connection. The survey queries participants about internal states that can only be captured by self-report such as mood, pain level, or loneliness, as well as life events that may affect activity data inference. The weekly survey data is structured in a forced-choice format composed of 13 questions (rate your pain on a 10-point scale, rate your mood, were you hospitalized or in an ER, did you have overnight visitors, did you change your medications, did you fall, were you injured, did you require more help or services, etc.) and typically takes less than five minutes to complete. Opportunity for free text information is also included. The weekly self-report survey also is used as a passive measure of potential cognitive decline, providing metrics such as general typing data (e.g., variation in number of mouse clicks or movement events), variation in the time to complete the survey, increased difficulty reporting accurate dates, or impairment markers in free text response [33,34].
