# Supplementary material for: An Ecologically Valid, Longitudinal, and Unbiased Assessment of Treatment Efficacy in Alzheimer Disease (the EVALUATE-AD Trial): Proof-of-Concept Study
Source: JMIR Res Protoc. 2020 May 27;9(5):e17603. doi: 10.2196/17603 (PMC7287724; doi:10.2196/17603)
Supplement: Multimedia Appendix 2 [file resprot_v9i5e17603_app2.docx]

**Participant ID: Date:**

**ORCATECH PARTICIPANT STUDY PERCEPTION QUESTIONNAIRE – Patient Version**

**General attitudes toward monitoring**

|  | Strongly Agree | Agree | Neutral | Disagree | Strongly Disagree |
| --- | --- | --- | --- | --- | --- |
| 1. I do not mind being monitored unobtrusively in my home. |  |  |  |  |  |
| 1. I do not mind being monitored as long as the data collected is useful for my doctor. |  |  |  |  |  |

1. What do you think about the sensors installed in your home?
2. Do you think often about the sensor system? Y / N
3. Did you have any visitors or family who asked about the sensors? Y / N
4. Did having the technologies in your home interfere with or change your daily routine? Y / N (if yes, how?)
5. Did your overall attitude towards the system change over time? Y / N (if yes, how?)

**Access to information from the sensor system**

|  | Strongly Agree | Agree | Neutral | Disagree | Strongly Disagree |
| --- | --- | --- | --- | --- | --- |
| 1. I do not care who has access to information from in-home activity or computer monitoring. |  |  |  |  |  |
| 1. I would want information about my activity sent to me if there was a change in my activity. |  |  |  |  |  |
| 1. I would want information about my activity sent to me if the changes suggest that I might have worsening of my memory and thinking. |  |  |  |  |  |

1. Would data from the sensors and wristwatch that show how active you are in your home have any usefulness for you? Y / N (if yes, how?)
2. Would data from the sensors and wristwatch that show how you are sleeping have any usefulness for you? Y / N (if yes, how?)
3. Would you like to see your activity and sleep information? Y / N
4. How often would you like to see the data about your own activities of daily living?
   1. Daily
   2. Weekly
   3. Monthly
   4. Other frequency:

**Attitudes towards sharing information from the sensor system with family or one's doctor**

|  | Strongly Agree | Agree | Neutral | Disagree | Strongly Disagree |
| --- | --- | --- | --- | --- | --- |
| 1. I would want information about my activity sent to a family member if the changes suggest that my memory and thinking were worsening. |  |  |  |  |  |
| 1. I would want information about my activity sent to my doctor if the changes my memory and thinking were worsening. |  |  |  |  |  |
| 1. I am willing to have information from activity monitoring shared with my family. |  |  |  |  |  |
| 1. I am willing to have information from activity monitoring shared with my doctor. |  |  |  |  |  |
| 1. I am willing to have information from my computer use shared with my family. |  |  |  |  |  |
| 1. I am willing to have information from my computer use shared with my doctor. |  |  |  |  |  |

**Privacy and Security Concerns**

|  | Very Concerned | Somewhat Concerned | Not Very Concerned | Not Concerned At All |
| --- | --- | --- | --- | --- |
| 1. I am concerned information could be given to people/organizations that do not have a right to it. |  |  |  |  |
| 1. I am concerned information could be given to people/organizations that would use it in a way that would harm you. |  |  |  |  |
| 1. I am concerned about privacy in relation to in-home activity monitoring. |  |  |  |  |
| 1. I am concerned about privacy in relation to monitoring of computer use. |  |  |  |  |

1. Do you have any general privacy or other concerns with the use of home based monitoring technology for health purposes? Y / N (if yes, please explain?)

**Wearable Technology**

|  | Strongly Agree | Agree | Neutral | Disagree | Strongly Disagree |
| --- | --- | --- | --- | --- | --- |
| 1. I did not mind wearing the activity monitoring wristwatch 24/7. |  |  |  |  |  |
| 1. The activity monitoring wristwatch did not bother me while I slept. |  |  |  |  |  |
| 1. The activity monitoring wristwatch was comfortable. |  |  |  |  |  |
| 1. I liked not having to charge the wristwatch. |  |  |  |  |  |

1. What do you think about the activity monitoring wristwatch?
2. Did wearing the watch change your activity pattern at all during the day? Y / N (if yes, how?)
3. Did wearing the watch interfere with your sleep? Y / N (if yes, how?)

**Driving**

|  | Strongly Agree | Agree | Neutral | Disagree | Strongly Disagree |
| --- | --- | --- | --- | --- | --- |
| 1. I did not mind having a driving sensor placed in my vehicle. |  |  |  |  |  |
| 1. I would like to have access to the data from the driving sensor. |  |  |  |  |  |

1. What do you think about the driving sensor?

**Overall Thoughts**

1. Are there any other thoughts you would like to share about the system or this research study?
2. Do you have any suggestions for what should be being done different in this study?
